# Vomiting in Neuromyelitis Optica: An Atypical Presentation

**DOI:** 10.7759/cureus.5225

**Published:** 2019-07-24

**Authors:** Syed M Zaidi, Aleena Mohib, Syed Asad Hasan Rizvi, Amara Zafar

**Affiliations:** 1 Internal Medicine, Civil Hospital Karachi, Dow University of Health Sciences, Karachi, PAK; 2 Medicine, Civil Hospital Karachi, Dow University of Health Sciences, Karachi, PAK; 3 Internal Medicine, Civil Hospital Karachi, Karachi, PAK

**Keywords:** devic's syndrome, neuromyelitis optica spectrum disorders, vomiting

## Abstract

Neuromyelitis Optica (NMO), also known as Devic’s disease, is a demyelinating disorder of the central nervous system (CNS) that majorly involves the optic nerves and the spinal cord. It is an idiopathic and an autoimmune disorder. The patient typically presents with symptoms pertaining to the eye or spinal cord, such as a decrease in visual acuity, visual field defects, pain in the eyes, loss of vision, numbness, and weakness of the limbs with or without bladder dysfunction. Vomiting, however, is an atypical presentation of this disorder. We report here a case of an 18-year-old female who presented to our tertiary care set-up with a one-month history of intractable non-bilious vomiting and dull epigastric pain. After going through several initial investigations and being discharged home, she returned after five days with complaints of intractable vomiting, double vision and inability to stand or walk. Later, the presence of anti-NMO antibodies led to the diagnosis of Neuromyelitis Optica Spectrum Disorder (NMOSD). Through this case, we highlight the importance of considering NMO in a patient presenting with intractable vomiting to enable prompt diagnosis and treatment of the disease, thus preventing further disability.

## Introduction

Neuromyelitis Optica (NMO), also known as Devic’s disease, is a disorder of the central nervous system (CNS). It is an idiopathic, autoimmune and demyelinating disease predominantly involving the optic nerves and the spinal cord [1]. Optic neuritis (dysfunction of the optic nerves) and transverse myelitis (dysfunction of the spinal cord) are the classic clinical manifestations of NMO and the most common presentation of the disease [2]. It was not known until recently that intractable vomiting was reported in the literature as a much rarer initial presentation of NMO. Here we report a case of an 18-year-old female who presented with intractable vomiting as the initial presenting complaint and was diagnosed with NMO a few days later. 

## Case presentation

An 18-year-old female was admitted to a tertiary care hospital in Karachi with a history of non-bilious vomiting of about 10-12 episodes per day and gradual onset of dull epigastric pain radiating to the back for the past one month. She also reported undocumented weight loss along with headache, dizziness and generalized weakness during this period. On examination at the time of presentation, her vitals were stable. There was mild epigastric tenderness on an abdominal examination. On CNS examination, higher mental functions were intact. Speech and gait were normal. Cranial nerves and sensory system were grossly intact. Signs of cerebellar disease and meningeal irritation were absent. Motor examination revealed a generalized decrease in tone and power of both upper and lower limbs with normal reflexes (++) and down-going plantar response. Examinations of all other systems were otherwise unremarkable.

Laboratory investigations revealed hemoglobin of 12.3 g/dL, white cell count of 11.4 x 10^9^ IU/L with 81% neutrophils, 12% lymphocytes and 2% monocytes. Platelet count was 476,000 IU/L. Blood urea nitrogen and creatinine levels were within the normal range. Serum electrolyte tests, however, showed low potassium levels of 1.8 mEq/L and low bicarbonate levels of 15 mEq/L. Results of liver function tests, clotting profile and urine detailed report were within the normal range. Urinary pH was, however, raised on three consecutive days (pH of 7, 7 and 8 on day 1, day 2 and day 3 respectively). Erythrocyte sedimentation rate (ESR) and C-reactive protein (CRP) levels were within the normal range. Serum amylase and lipase levels were within the normal limit as well. Stool test for Helicobacter pylori antigen was negative. Tests for Hepatitis B surface antigen (HBsAg) and anti-Hepatitis C virus (anti-HCV) were also negative. Serum cortisol levels were slightly decreased (8.23 microgram/dL).

Findings of electrocardiography (ECG), chest x-ray, ultrasound abdomen and computed tomography (CT) scan abdomen with contrast were unremarkable. Results of endoscopy also turned out to be unremarkable. Repeat potassium and bicarbonate levels showed persistently low levels. Considering the possibility of distal renal tubular acidosis (RTA), an autoimmune profile was performed. It revealed positive anti-nuclear antibody (ANA), negative anti-double-stranded DNA (anti-dsDNA), negative anti-ribonucleoprotein (anti-RNP), negative anti-smith antibody (anti-Sm), negative anti-Sjögren's syndrome type A antibody (anti-SS-A), negative anti-Sjögren's syndrome type B antibody (anti-SS-B), negative anti-ScL-70 antibody (autoantibodies against topoisomerase I) and negative anti-Jo-1 antibody (autoantibodies against histidyl-tRNA synthetase). Her complement levels showed slightly low C3 levels of 0.86g/L, while C4 levels were within the normal range.

The patient was given sodium bicarbonate tablets and her response to the treatment was observed. She responded well, as indicated by her repeat potassium levels of 3.5 mEq/L, 4.0 mEq/L and 4.2 mEq/L on three different occasions. She was discharged home with advice to follow up.

Five days after her discharge, she presented in the outpatient clinic with complaints of intractable vomiting, double vision (on looking towards the left side by confrontation method) and inability to stand and walk. The relative afferent pupillary defect was absent. Bilateral fundi were unremarkable. On cranial nerve examination, the patient was unable to adduct her right eye. Other extraocular muscles were intact on the right side. All other movements of extraocular muscles were intact. Motor examination revealed increased tone and reflexes bilaterally in upper and lower limbs. Power was reduced bilaterally in upper and lower limbs (3/5). An up-going plantar response was detected. The sensory system was grossly intact. Signs of cerebellar disease and meningeal irritation were absent.

**Figure 1:**
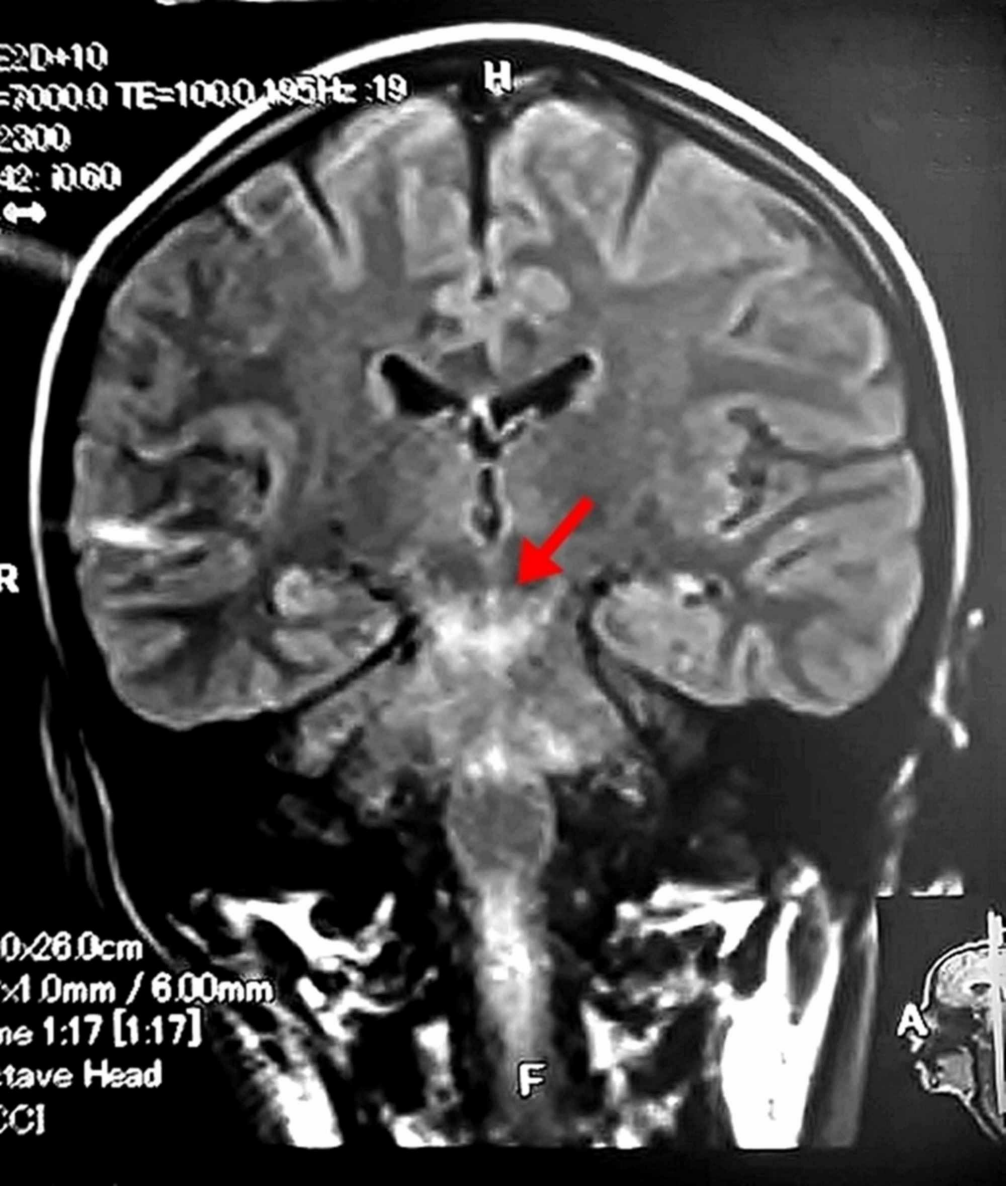
MRI of the brain showing abnormal signal intensity changes seen in the midbrain and pons extending into the periaqueductal region

Magnetic resonance imaging (MRI) of the brain and spine was performed. As shown in figure 1, it revealed abnormal signal intensity changes in the midbrain and pons extending into the periaqueductal region and also inferiorly into the cranial aspect of the cervical spinal cord up to the C5 segment. These were hyperintense on T2 weighted images and fluid-attenuated inversion recovery (FLAIR) images, and iso-intense on T1 weighted images. No evidence of an intracranial bleed, mass or gross area of infarction was found. Findings were suggestive of demyelination or a neurodegenerative disorder.

To investigate for transverse myelitis, MRI of dorso-lumbar spine was performed, which was normal. The visual evoked potential was normal in both eyes. A test for anti-NMO antibodies was then performed, which turned out to be positive. Based on these findings, the patient was diagnosed with neuromyelitis optica spectrum disorder (NMOSD).

The patient was treated with 1 gm high dose intravenous corticosteroids for five days, which resulted in resolution of vomiting and ocular symptoms. After achieving remission, the treatment was slowly transitioned to oral corticosteroids in a dose of 1mg/kg/day. Azathioprine was added in a dose of 3mg/kg/day. The patient is being followed up to date and remains relapse-free.

## Discussion

NMO, also known as Devic’s disease, is an idiopathic, autoimmune demyelinating disorder with a predilection for the optic nerves and spinal cord [1]. The typical clinical presentation of NMO comprises of symptoms pertaining to the dysfunction of optic nerves and spinal cord, known as optic neuritis and transverse myelitis respectively. These are two of the characteristic clinical syndromes of NMO [2]. Wingerchuk et al. reported isolated optic neuritis or myelitis as the presentation in 90% of the patients with relapsing NMO [1]. A more recent review corroborated these findings [3]. Optic neuritis has been reported to be the initial event of NMO in over half of the patients presenting with decreased visual acuity, visual field defects, and complaints of eye pain and severe loss of vision [4]. However, the population of patients which initially presents with isolated myelitis typically complains of numbness and weakness of the limbs or trunk with or without associated bowel and bladder dysfunction [5].

Owing to the similarities in clinical presentation, up until recently, NMO was considered part of the same clinical entity as Multiple Sclerosis (MS) and was often misdiagnosed. With further understanding of the subject, NMO is now believed to have distinct pathogenesis involving the development of NMO-IgG autoantibodies against the protein aquaporin 4, found abundantly on the cell membranes in the CNS and spinal cord [6]. The presence of these antibodies can be used as a biomarker to clearly distinguish NMO from MS [6].

A much rarer presentation of NMO is intractable vomiting, which until recently was infrequently reported in the literature. A study done at Mayo Clinic reported only 12% of patients with NMO presented with intractable vomiting [7]. Furthermore, a case was reported where a 17 year old female presenting with intractable nausea and vomiting. She was finally diagnosed as a case of NMO after weeks of investigation, similar to our case [8]. Jin X et al. reported 12 cases of NMO with a similar presentation, of which 83% were aquaporin-4 antibody positive [9], while another study reported 14 cases of NMO with similar initial manifestations as well [10]. More reports can be found in the literature from Asia, including cases from India and China [11,12]. Contrary to our case, intractable hiccups were also a prominent symptom of NMO in other cases [13]. A similar case from India [14] was reported which was also in a young female like in our case, corroborating the fact that the prevalence of NMO is highest among females [3].

Another notable feature is the onset of optic neuritis in the course of the disease, which in our case was seen to be a few weeks after the initial complaint of vomiting, without the presence of any other prior, overt neurological symptoms. This is consistent with the past cases reported in the literature [8,12]. Despite many cases being reported from the region of Asia, particularly many from India [11], the case in hand is the solitary case of NMO presenting with intractable vomiting to be reported from Pakistan and can be considered a stepping-stone for this under-reported subject.

The likely cause of initial manifestation of NMO with intractable vomiting, nausea and/or hiccups has been associated with a rather uncommon aspect of the disease called area postrema syndrome [11]. Area postrema syndrome is defined as an episode of hiccups or nausea and vomiting due to pathology in the region of area postrema, with no other known cause or explanation [15]. It has now been included as one of the core clinical characteristics in the revised diagnostic criteria for NMO [16]. Area postrema is located in the floor of the fourth ventricle in the dorsal medulla, and together with the nucleus tractus solitarius, forms the control center of emesis. The protein aquaporin 4 is more abundantly expressed in the area postrema and the fourth ventricle floor relative to other areas of the brain, and since NMO-IgG antibodies act against this specific protein, these aquaporin 4-rich areas are preferentially involved in NMO [17]. A study by Popescu et al. stated that the likelihood of NMO presenting with nausea or vomiting is about 16 times higher with lesions in area postrema [18].

Since vomiting and nausea are quite non-specific symptoms and usually pertain to gastrointestinal tract diseases, physicians usually do not associate these symptoms with a neurological pathology until everything else on their list of differential diagnoses is ruled out. This leads to a delay in diagnosis or a misdiagnosis. A Chinese study reported misdiagnoses of 11 patients of NMO, with the majority being diagnosed as gastroesophageal reflux disease [10]. Furthermore, symptoms like vomiting most commonly present acutely in the ER. In most case reports including ours, these are managed symptomatically by ER physicians or are referred to the gastroenterology department. A case report by Apiwattanakul et al. reported 75% of cases of NMO presenting with vomiting were initially evaluated by gastroenterology department [19], which is in agreement with multiple other studies in the literature.

Taking into consideration the growing frequency of reports of NMO with initial manifestations of intractable vomiting and/or nausea, the importance of considering NMO in the differential diagnoses during the management of such symptoms cannot be overstated. This may reduce the barrage of unnecessary investigations, which are usually carried out, possibly due to the absence of overt or early neurological symptoms in such cases. More importantly, the time taken to reach the diagnosis can be significantly shortened, enabling prompt treatment of the disease and preventing further disability. Saving resources and time of the patient in the management of rare diseases like NMO may be of special importance for countries like Pakistan, where the health-care system is mostly underdeveloped.

## Conclusions

Through this case report, we highlight the importance of considering the possibility of a neurological pathology, including Neuromyelitis Optica Spectrum Disorder, to be the cause of intractable vomiting. This can help to ensure timely diagnosis and prompt treatment of the patient.
